# Effect of prolonged stress on the adrenal hormones of individuals with irritable bowel syndrome

**DOI:** 10.1186/s13030-015-0031-7

**Published:** 2015-01-23

**Authors:** Nagisa Sugaya, Shuhei Izawa, Keisuke Saito, Kentaro Shirotsuki, Shinobu Nomura, Hironori Shimada

**Affiliations:** Department of Epidemiology and Public Health, Graduate School of Medicine, Yokohama City University, 3-9 Fukuura, Kanazawa-ku, Yokohama, Kanagawa 236-0004 Japan; Health Administration and Psychosocial Factor Research Group, National Institute of Occupational Safety and Health, 6-21-1 Nagao, Tama-ku, Kawasaki-shi, Kanagawa 214-8585 Japan; Department of Children Education, Tokai University Junior College, 101 Miyamaecho, Aoi-ku, Shizuoka 420-8511 Japan; Faculty of Human Sciences, Musashino University, 3-3-3 Ariake, Koto-ku, Tokyo 135-8181 Japan; Faculty of Human Sciences, Waseda University, 2-579-15 Mikajima, Tokorozawa, Saitama 359-1192 Japan

**Keywords:** Irritable bowel syndrome, Cortisol, Dehydroepiandrosterone, Saliva, Prolonged stress

## Abstract

**Background:**

The purpose of this study was to investigate the effect of prolonged stress on the salivary adrenal hormones (cortisol, dehydroepiandrosterone [DHEA], DHEA-sulfate [DHEA-S]) of individuals with irritable bowel syndrome (IBS).

**Methods:**

The participants were female college students, including 10 with IBS and 16 without IBS (control group), who were scheduled for a 2-week teaching practice at a kindergarten. Participants were asked to collect saliva for determining adrenal hormones immediately and 30 min after awakening and before sleep, 2 weeks before the practice, the first week of the practice, the second week of the practice, and a few days after the practice.

**Results:**

Regarding cortisol/DHEA ratio, significantly increased levels were found during the first week of the practice, and a significant interaction between group and time was found; the ratio at 30 min after awakening in the IBS group was higher than that in the control group. For the other adrenal hormone indexes, no significant differences due to the presence of IBS were found.

**Conclusions:**

Individuals with IBS showed an elevated cortisol/DHEA ratio after awakening compared with individuals without IBS, and the elevated ratio peaked under the prolonged stress. The present study suggests that the cortisol effect is dominant in individuals with IBS under prolonged stress.

## Introduction

Irritable bowel syndrome (IBS) is a functional gastrointestinal disorder characterized by persistent chronic abdominal pain and disturbance of bowel movements. It is a typical digestive system psychosomatic disorder that is exacerbated by stress. Previous research has shown exaggerated colonic motor response to laboratory stress in IBS patients [1,2].

Recently, the brain-gut axis in IBS has attracted much attention, and previous studies have shown interrelationships between the hypothalamic–pituitary–adrenal (HPA) axis, gut-associated immune tissues, and the enteric nervous system under stress. Parameters of the HPA axis include the corticotropin-releasing hormone (CRH) secreted by the hypothalamic paraventricular nucleus, the adrenocorticotrophic hormone (ACTH) secreted by the anterior pituitary, and the cortisol secreted by the zona fasciculata of the adrenal cortex. Psychosocial stressors activate the HPA axis including cortisol [3]. Cortisol induces the mucosal immune system to activate a Th2 response, and as a result, mast cell numbers are increased [4,5]. Mast cells in the gut produce and release abundant histamine, serotonin, and proteases and cause more excitation of the primary afferent neurons in IBS patients than in healthy controls [6-8]. Thus, increased cortisol is reported to contribute to the deterioration of abdominal symptoms in IBS through changes in gut-associated immune tissues and the enteric nervous system.

Characteristics of HPA axis parameters under various situations have been revealed in previous studies. IBS patients were reported to secrete more CRH than healthy control individuals [9], and to have accelerated rates of ACTH [10] and cortisol [11] secretion in response to CRH, while another study reported that there was no difference in cortisol secretion between IBS and control individuals [10]. Under an acute psychological stress task, individuals with IBS were reported to show no difference in the cortisol response compared with individuals without IBS [12-15], although individuals with IBS displayed higher CRH and ACTH responses compared with individuals without IBS [14]. On the other hand, some studies reported that basal and morning cortisol levels were elevated in IBS patients compared with healthy controls [11,16,17]. These studies indicate complex relationships between IBS and the HPA axis, and differences of cortisol secretion between situations in IBS.

Although the adrenal hormone response under acute psychological stress in individuals with IBS has been assessed in previous research, the response to prolonged stress (lasting certain periods of time, e.g., a few weeks or months) has not been defined. Cortisol levels have been reported to change in prolonged stress [18,19]. A prospective controlled study [20] also found an increase of overall postawakening salivary cortisol concentrations in students participating in a major examination compared with matched control students before, during, and after the examination. Because our previous study [15] focused on adrenal hormones in individuals with IBS only under acute psychological stress and used the Trier Social Stress Test [21] including a five-minute speech and a five-minute mental arithmetic task, we need to consider these indexes in IBS under different stressful situations. Because IBS tends to be a long-lasting condition, assessment of the HPA axis under a prolonged stress situation may be a suitable study design for the clinical picture of IBS.

Moreover, dehydroepiandrosterone (DHEA) may be an important and useful adrenal hormone to understand the pathophysiology of IBS. DHEA is secreted from the zona reticularis. ACTH is thought to be the main secretagogue for DHEA. DHEA is thought to affect immunological function in a manner opposite to that of cortisol; DHEA increases Th1 cytokines [22-24]. Administration of DHEA reduced activity in the amygdala, which activates the HPA axis [25]. DHEA and cortisol/DHEA ratio were also reported to increase under acute and prolonged psychological stress [19,26]. Moreover, DHEA is converted into DHEA-sulfate (DHEA-S) by a sulfotransferase and accumulates within a relatively short time after secretion. Thus, DHEA-S is the major circulating form and is further converted to DHEA by a steroid sulfatase in the tissue. Previous research suggests that lower DHEA-S concentrations in plasma or serum are related to stronger perceived stress [27]. Our previous studies revealed that a low 8-day average DHEA-S level at awakening was associated with frequent bowel movements, loose stools, and long bouts of severe abdominal pain [28], and that individuals with IBS had lower DHEA-S levels under acute psychosocial stress, which could cause the effects of cortisol to dominate [15]. Additionally, DHEA-S/DHEA ratio was also reported to be associated with psychological stress or psychiatric diseases [29]. Therefore, assessment of not only cortisol but also DHEA and DHEA-S may provide clues regarding the underlying cause of aggravation of IBS symptoms when considering psychological influence on IBS.

Therefore, the purpose of the present study was to investigate the effect of prolonged stress on the adrenal hormones of individuals with IBS. The present study focused on the differences in the adrenal hormone response (cortisol, DHEA, DHEAS, cortisol/DHEA ratio, and DHEA-S/DHEA ratio) to prolonged stress of individuals with and without IBS. We hypothesized that individuals with IBS would show a higher cortisol level or lower DHEA and DHEA-S levels under prolonged stress.

## Methods

### Participants

We distributed leaflets explaining this experiment to university students and held a briefing session for interested students, each of whom provided written informed consent prior to participating. First, we collected data from 33 participants who scheduled a 2-week teaching practice at a kindergarten. Because the present study focused primarily on the balance between cortisol and DHEA, we excluded 5 samples that contained much missing data for either cortisol or DHEA and 2 samples of relatively older participants (29 and 35 years) as DHEA was reported to decrease with aging [30]. Valid data were obtained from 26 participants. The mean (standard deviation) age and body mass index (BMI) were 18.73 (0.45) years and 21.10 (2.94) kg/m^2^, respectively. The participants included 16 control group members who did not meet the criteria for IBS and 10 individuals with IBS (diarrhea-predominant IBS: 1, constipation-predominant IBS: 3, other types [participants who did not meet criteria for diarrhea-predominant IBS or constipation-predominant IBS but who met criteria for both types]: 6). All the participants were non-smokers and none used medications (including oral contraceptives) or dietary supplements known to affect HPA axis activity. Written informed consent was obtained before commencement of the study, and the study was approved by the Ethics Committee of Waseda university.

### Questionnaires

#### Rome II modular questionnaire (only items related to IBS), Japanese version

The Rome II diagnostic criteria for gastrointestinal disorders are used widely to define IBS. Using the Rome II modular questionnaire (RMQ) [31,32], the presence of IBS was determined if the participant had abdominal pain or discomfort during at least 3 weeks (at least once per week) in the previous 3 months and 2 of the 3 following symptoms: (1) pain or discomfort that improves or stops after a bowel movement, (2) a change in the number of bowel movements when the pain or discomfort starts, and (3) either softer or harder stools than usual when the pain or discomfort starts.

#### Red-flag items

Seven red-flag items, based on the guidelines for IBS of the American Gastroenterological Association, were used to distinguish organic from functional gastrointestinal diseases. Individuals reporting one or more of the following items were excluded from this study: Drastic weight loss, the participant’s or a family history of organic bowel disease, history of digestive surgery, awakening by abdominal pain during sleep, fever or arthralgia, blood in the stool, and anemia.

#### Perceived stress scale, Japanese version

The Perceived Stress Scale (PSS) [33,34] was designed to measure the degree to which the situations in one’s life are appraised as stressful. The PSS includes 14 items, each of which is rated from 0 to 4. In this study, we assessed perceived stress experienced during the preceding 2 or 3 days. The internal reliabilities (α coefficients) ranged from 0.82 to 0.89.

### Determination of adrenal hormone levels

The saliva samples for determining cortisol, DHEA, and DHEA-S levels were collected by the passive drool method using a short plastic straw and a collection vial (for details, see Granger et al., [35]). This collection method was recommended by previous studies because the use of a cotton swab, such as Salivette, interferes with salivary immunoassay analysis for DHEA [36]. Saliva samples were stored at −20°C after sampling and centrifuged at 3000 rpm for 15 min before determination of adrenal hormone levels. The concentrations of cortisol, DHEA, and DHEA-S in the saliva were determined by enzyme immunoassay using an EIA kit (Cortisol and DHEA: Salimetrics LLC, USA; DHEA-S: DiaMetra, Italy). For cortisol, the inter-assay and intra-assay variations were below 6.4% and 3.7%, respectively; for DHEA, they were below 8.5% and 5.8%, respectively, and for DHEA-S, they were below 8.9% and 4.8%, respectively.

### Procedure

A detailed procedure of the present study can be found in our previous paper [19]. Saliva samples were collected at 4 time points: 2 weeks before the teaching practice (Day 1), the first week of the teaching practice (approximately the third day of the practice, Day 2), the second week of the practice (approximately the tenth day of the practice, Day 3), and a few days after the practice (Day 4). Saliva samples were collected on a weekday. The participants were asked to collect their saliva at awakening, 30 min after awakening, and at bedtime each day. They were instructed to refrain from eating, drinking, or brushing their teeth for 30 min after awakening and 1 h before bedtime. Participants completed the RMQ and red-flag items before the start of the teaching practice. They completed the PSS and an item about the degree of abdominal pain, rating it from 0 (not at all) to 10 (very severe) at bedtime on each sample day. In addition, they recorded the time of going to bed, time of awakening, time of collecting each saliva sample, stress at awakening rated from 1 (not at all) to 4 (very severe), and the last menstrual day and intervals of menstrual cycles for estimation of menstrual phase (for details, see Isowa et al., [37]) on each sample day. Moreover, on Day 2 and Day 3, they were questioned about their experiences with the special practices (setting and implementing their own agendas responsibly by themselves on that day) at the kindergarten during the preceding 7 days, which was considered an acute stressor.

### Statistical analysis

For the analysis of adrenal hormone data, the concentrations of cortisol, DHEA, and DHEA-S were square-root transformed because the Kolmogorov–Smirnov test indicated that the distributions were skewed. The DHEA-S level was adjusted for saliva flow (measured concentration).

Data analysis was performed using SPSS 20.0 software (SPSS, Inc., Chicago, IL). The *t* test was applied to compare age and BMI before the experiment according to the presence or absence of IBS. A linear mixed model was applied to compare the PSS score, awakening time, sleeping hours, and degree of abdominal pain according to the presence or absence of IBS and day. We conducted correlation analyses (Pearson correlation) to determine any associations between adrenal hormones (cortisol, DHEA, DHEA-S, cortisol/DHEA ratio, and DHEA-S/DHEA ratio) and PSS scores on each sampling day and time. Multiple testing corrections were performed by Bonferroni tests with p < 0.0083 (=0.05/60). A linear mixed model was applied to analyze the effects of the presence of IBS and the particular time point on adrenal hormone levels; day (Day 1–Day 4), time point (at awakening, 30 min after awakening, and at bedtime), and group (IBS or control) were treated as fixed effects, and awakening time, menstrual phase (luteal or follicular phase), stress at awakening (reflecting anticipation of the day at the kindergarten), and acute stressor (special practice day or normal practice day) were treated as covariates. Post hoc comparisons between days (Day 1–Day 4) and between awakening, 30 min after awakening, and bedtime were accomplished using Shaffer’s sequentially rejective multiple test procedure.

## Results

### Characteristics of the participants

The mean age in the IBS and control groups was 18.90 (0.32) years and 18.63 (0.50) years, respectively, and the mean BMI in the IBS and control groups was 20.46 (3.11) kg/m^2^ and 21.48 (2.87) kg/m^2^, respectively (Table 1). There were no significant differences between the groups in age or BMI.

**Table 1:** **Characteristics of participants**

	**IBS (N = 10)**	**Control (N =16)**	***p***
Age (years)	18.90 ± 0.32	18.63 ± 0.50	0.10
BMI (kg/m^2^)	20.46 ± 3.11	21.48 ± 2.87	0.42
PSS score Day 1	26.40 ± 5.13	24.67 ± 4.08	Interaction: 0.95 Group: 0.39 Day: <0.0001
Day 2	31.70 ± 7.92	29.67 ± 7.53	
Day 3	29.44 ± 6.97	27.19 ± 4.78	
Day 4	24.67 ± 7.68	23.69 ± 5.75	
Abdominal pain Day 1	0.50 ± 1.08	0.19 ± 0.75	Interaction: 0.90 Group: 0.01 Day: 0.74
Day 2	0.40 ± 0.97	0.00 ± 0.00	
Day 3	0.89 ± 2.03	0.28 ± 1.13	
Day 4	0.78 ± 2.33	0.00 ± 0.00	

Mean ± standard deviation.

Age and BMI: No significant difference between groups.

PSS: Day 2 and Day 3 > Day 1 and Day 4 (*F* [3.00, 68.72] = 10.01, *p* < 0.0001); no significant interaction and group difference.

Abdominal pain: IBS group > control group (*F* [1.00, 24.3] = 7.00, *p* = 0.01); no significant interaction or difference among days.

The linear mixed model for the PSS score, awakening time, and sleeping hours showed significant main effects of day (PSS: *F* [3.00, 68.72] = 10.01, *p* < 0.0001; awakening time: *F* [3.00, 70.71] = 19.27, *p* < 0.0001; sleeping hours: *F* [3.00, 68.89] = 21.21, *p* < 0.0001, Table 1). The scores of PSS on Day 2 and Day 3 were higher than those on Day 1 and Day 4. Awakening times on Day 2 and Day 3 were earlier than those on Day 1 and Day 4, and that on Day 1 was earlier than that on Day 4. Sleeping hours on Day 2 and Day 3 were shorter than those on Day 1 and Day 4. No main effects of group or interactions in the PSS score, awakening time, and sleeping hours were found.

The linear mixed model for degree of abdominal pain showed a significant main effect of group (*F* [1.00, 24.3] = 7.00, *p* = 0.01, Table 1), and the degree of abdominal pain in the IBS group was higher than that in the control group. No main effect of day or interaction in degree of abdominal pain was found.

Regarding correlation analyses between adrenal hormones and the PSS score on each sample day and time, on Day 4, the DHEA-S and DHEA-S/DHEA ratio was positively correlated with the PSS score at awakening (DHEA-S: *r* = 0.50, *p* = 0.01; DHEA-S/DHEA ratio: *r* = 0.48, *p* = 0.03, uncorrected). However, these significant correlations disappeared after a Bonferroni correction for multiple testing.

### Comparison of adrenal hormone levels between IBS cases and controls during the experimental period

#### Increase of cortisol levels in IBS cases and controls under prolonged stress

The linear mixed model for cortisol concentration showed significant main effects of day (*F* [3.00, 250.28] = 5.16, *p* = 0.002) and time (*F* [2.00, 238.76] = 171.86, *p* < 0.0001), no significant main effect of group, and no significant interactions among IBS, day, and time (Table 2).

**Table 2:** **Cortisol, DHEA, DHEA-S, and DHEA-S/DHEA ratio**

		**Cortisol**				**DHEA**			
		**IBS**		**Control**		**IBS**		**Control**	
**Day 1**	**Time 1**	2.93	(0.29)	2.68	(0.24)	1.09	(0.08)	1.04	(0.06)
	**Time 2**	3.82	(0.30)	3.87	(0.24)	1.08	(0.08)	1.14	(0.06)
	**Time 3**	1.44	(0.29)	1.08	(0.25)	0.74	(0.08)	0.81	(0.06)
**Day 2**	**Time 1**	3.24	(0.30)	3.50	(0.24)	1.19	(0.08)	1.09	(0.07)
	**Time 2**	4.64	(0.30)	3.65	(0.24)	1.10	(0.08)	1.12	(0.06)
	**Time 3**	1.85	(0.30)	2.06	(0.25)	0.80	(0.08)	0.77	(0.06)
**Day 3**	**Time 1**	3.19	(0.32)	3.33	(0.23)	1.16	(0.08)	1.25	(0.06)
	**Time 2**	4.52	(0.32)	4.22	(0.23)	1.11	(0.08)	1.26	(0.06)
	**Time 3**	1.66	(0.33)	1.94	(0.26)	0.73	(0.08)	0.80	(0.06)
**Day 4**	**Time 1**	2.56	(0.31)	3.13	(0.25)	0.94	(0.09)	1.00	(0.07)
	**Time 2**	3.28	(0.31)	3.19	(0.25)	0.95	(0.08)	1.04	(0.06)
	**Time 3**	1.15	(0.33)	1.64	(0.25)	0.65	(0.08)	0.70	(0.06)
		**DHEA-S**				**DHEA-S/DHEA**			
		**IBS**		**Control**		**IBS**		**Control**	
**Day 1**	**Time 1**	3.85	(0.44)	3.48	(0.36)	3.85	(0.53)	3.38	(0.44)
	**Time 2**	2.23	(0.45)	2.93	(0.36)	2.23	(0.55)	2.55	(0.44)
	**Time 3**	2.80	(0.45)	2.43	(0.36)	4.07	(0.55)	3.07	(0.44)
**Day 2**	**Time 1**	3.13	(0.45)	3.52	(0.36)	2.58	(0.55)	3.10	(0.49)
	**Time 2**	2.26	(0.45)	2.56	(0.36)	2.04	(0.55)	2.44	(0.44)
	**Time 3**	1.76	(0.45)	2.26	(0.36)	2.32	(0.55)	3.16	(0.44)
**Day 3**	**Time 1**	3.77	(0.47)	3.86	(0.36)	3.52	(0.58)	3.25	(0.44)
	**Time 2**	1.70	(0.47)	2.23	(0.36)	1.52	(0.58)	1.84	(0.43)
	**Time 3**	2.01	(0.45)	2.59	(0.36)	3.00	(0.55)	3.42	(0.44)
**Day 4**	**Time 1**	2.62	(0.48)	3.29	(0.37)	2.61	(0.62)	3.50	(0.46)
	**Time 2**	1.95	(0.46)	3.20	(0.37)	2.22	(0.57)	3.20	(0.45)
	**Time 3**	2.14	(0.45)	2.47	(0.37)	3.51	(0.55)	3.67	(0.45)

Mean (standard error).

N = 26 (IBS group: 10, control group: 16).

DHEA, dehydroepiandrosterone; DHEA-S, DHEA-sulfate; IBS, irritable bowel syndrome.

The post hoc test for day indicated that cortisol levels on Day 2 and Day 3 were higher than those on Day 1 and Day 4.

The post hoc test for time indicated that cortisol level at awakening was significantly higher than that at bedtime and that cortisol level 30 min after awakening was significantly higher than those at awakening and bedtime.

#### Increase of DHEA levels in IBS cases and controls under prolonged stress

The linear mixed model for DHEA concentration showed significant main effects of day (*F* [3.00, 252.67] = 4.00, *p* = 0.008) and time (*F* [2.00, 242.70] = 90.13, *p* < 0.0001), no significant main effect of IBS, and no significant interactions among IBS, day, and time (Table 2).

The post hoc test for day indicated that the DHEA level on Day 3 was significantly higher than that on Day 4, but a significantly high level of DHEA on Day 2 was not found.

The post hoc test for time indicated that DHEA levels at awakening and 30 min after awakening were significantly higher than that at bedtime.

#### No change of DHEA-S levels in IBS cases and controls under prolonged stress

The linear mixed model for DHEA-S concentration showed a significant main effect of time (*F* [2.00, 246.69] = 31.32, *p* < 0.0001), no significant main effects of group and day, and no significant interactions among IBS, day, and time (Table 2).

The post hoc test for time indicated that the DHEA-S level at awakening was significantly higher than those at 30 min after awakening and bedtime.

#### Increase of cortisol/DHEA ratio in IBS cases and controls and increased response of cortisol/DHEA ratio after awakening in IBS cases

**Figure 1:**
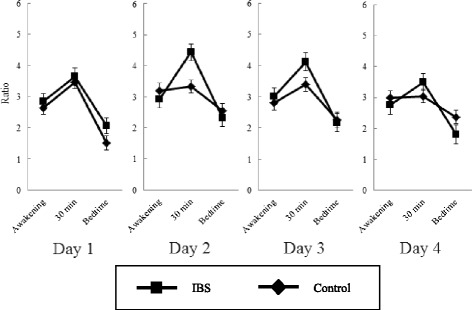
**Comparison of the transitions of the cortisol/DHEA ratio between the IBS group and the control group. N = 26 (IBS group, 10; control group, 16)**. Interaction between IBS and time (*F* [2.00, 231.79] = 6.81, *p* = 0.001): IBS > Control (30 min after awakening). Effect of day (*F* [3.00, 241.65] = 3.03, *p* = 0.03): Day 2 > Day 1. Error bars denote standard errors.

The linear mixed model for cortisol/DHEA ratio showed significant main effects of day (*F* [3.00, 241.65] = 3.03, *p* = 0.03) and time (*F* [2.00, 231.64] = 94.51, *p* < 0.0001), and a significant interaction between IBS and time (*F* [2.00, 231.79] = 6.81, *p* = 0.001) but no significant interactions between IBS and day or among IBS, day, and time (Figure 1).

The post hoc test for day indicated that the cortisol/DHEA ratio on Day 2 was significantly higher than that on Day 1.

The post hoc test for time indicated that the cortisol/DHEA ratio at awakening was significantly higher than that at bedtime, and that the ratio at 30 min after awakening was significantly higher than those at awakening and bedtime.

Interaction between IBS and time indicated that the cortisol/DHEA ratio at 30 min after awakening was significantly higher than those at awakening and bedtime, that the cortisol/DHEA ratio at awakening was significantly higher than that at bedtime, in both the IBS and control groups, and that the cortisol/DHEA ratio at 30 min in the IBS group was higher than that in the control group.

#### No change of DHEA-S/DHEA ratio in IBS cases and controls under prolonged stress

The linear mixed model for DHEA-S/DHEA ratio concentration showed a significant main effect of time (*F* [2.00, 240.01] = 17.95, *p* < 0.0001), no significant main effects of group and day, and no significant interactions among IBS, day, and time (Table 2).

The post hoc test for time indicated that DHEA-S/DHEA ratios at awakening and bedtime were significantly higher than that at 30 min after awakening.

## Discussion

The present study focused on the differences in the adrenal hormone response to prolonged stress of individuals with and without IBS. A significant interaction between group and time was found only in the cortisol/DHEA ratio. The cortisol/DHEA ratio at 30 min after awakening in the IBS group was higher than that in the control group for the entire experimental period, and both groups showed significant increases in the cortisol/DHEA ratio from 2 weeks before to the first week of the teaching practice; the elevated cortisol/DHEA ratio after awakening in individuals with IBS peaked under the prolonged stressful situation. Other adrenal hormones did not show any significant interaction, although there were significant effects of day for cortisol and DHEA and significant effects of time for cortisol, DHEA, DHEA-S, and DHEA-S/DHEA ratio. These results indicate that the prolonged stressor used in the present study was sufficient stimulus to influence adrenal hormones. Additionally, the scores of perceived stress increased during the teaching practice. Thus, the teaching practice acted as a prolonged stressful situation for participants in the present study, and we could appropriately evaluate characteristics of adrenal hormone secretions in individuals with IBS under prolonged stress. A possible explanation for the finding of no difference of perceived stress between the groups is that the participants of the present study did not use medications, including psychotropic agents and antidepressants, known to affect HPA axis activity and were unlikely to exhibit comorbid psychiatric diseases. Additionally, because significant correlations between the PSS score and adrenal hormones were not found, the degree of perceived stress did not show a simple linear relationship with adrenal hormones under prolonged stress.

The most noteworthy point of the present study is that individuals with IBS showed a higher cortisol/DHEA ratio at 30 min after awakening than individuals without IBS during the experimental period, although there were no significant interactions between IBS and another independent variable (day or time) or main effects of IBS in other adrenal hormone parameters. The cortisol/DHEA ratio of both individuals with and without IBS increased during the teaching practice. While the cortisol/DHEA ratio increased during the prolonged stress event regardless of the presence of IBS, the change in the cortisol/DHEA ratio of individuals with IBS was more remarkable in relation to individuals without IBS, regardless of date. Thus, cortisol effects might dominate in individuals with IBS when they are exposed to prolonged stress, and this could contribute to the onset and deterioration of IBS. However, the degree of abdominal pain in the IBS group did not change during the experimental period. However, the lack of change of abdominal pain might have been due to the question regarding the degree of abdominal pain being asked the day before, and we should interpret the cortisol effect on onset and deterioration of IBS cautiously. Given no effect of IBS on the cortisol/DHEA ratio under acute stress in our previous study [15], we think that the presence of IBS might affect the cortisol/DHEA ratio not under acute stress but under prolonged stress. Additionally, the effect of prolonged stress on the cortisol/DHEA ratio did not last until the end of the teaching practice. We think that this result may reflect not a normalization of adrenal hormone secretion but the increase of DHEA level on Day 3. Although the primary secretagogue for cortisol and DHEA is ACTH, cortisol is synthesized by 17-α-hydroxylase activity and DHEA is synthesized by the 17,20-lyase activity of cytochrome P450c17 [38,39]. We speculate that individuals with IBS have different enzyme amounts or functions or different functions of the zona fasciculata and zona reticularis compared with individuals without IBS. The effects of these differences by the presence of IBS on cortisol/DHEA ratio become prominent under prolonged stress conditions. Research on the detailed physical mechanism of the relationship between adrenal hormones and prolonged stress in IBS is worth attention.

On the other hand, regarding cortisol, the results of the present study differed from those of a previous study [40]. The present study revealed main effects of only day and time for cortisol; there was no significant main effect of IBS or interaction, while Suárez-Hitz et al. [40] reported that women with IBS showed a blunted cortisol awakening response despite a high cortisol level immediately after awakening. The ratio of IBS subtypes in this previous study (diarrhea: 22, constipation: 14, mix: 20) differed from that of the present study. Moreover, the participants of this previous study [40] included many individuals who had experienced emotional abuse, but we did not consider any experience of emotional abuse in the present study. Notably, some previous studies reported a relationship between abuse experiences and blunted cortisol response [41], as well as a higher prevalence of a history of abuse in individuals with IBS [42,43]. If the presence of abuse experiences and subtype of IBS are taken into consideration in future study design, a distinct effect of the presence of IBS may be found.

The present study identified differences and similarities of DHEA and DHEA-S under a prolonged stress situation compared to our previous study using an acute stress test [15]. Our previous study reported that there was no IBS-related difference in DHEA level under acute stress, and that individuals with IBS showed lower DHEA-S and DHEA-S/DHEA ratio throughout the acute stress experiment [15]. In the present study, the results of DHEA, DHEA-S, and DHEA-S/DHEA ratio revealed only circadian rhythms and no IBS-specific characteristics. Low DHEA-S levels in individuals with IBS could be unique to an acute stressful situation. On the other hand, the lower DHEA-S level under acute stress in the previous study [15] and the higher cortisol/DHEA ratio in individuals with IBS under prolonged stress in the present study may indicate a common finding of cortisol dominance under stressful situations in IBS. However, although both the present study using prolonged stress and the previous study using acute stress suggest that individuals with IBS show more prominent effects of cortisol under stressful situations than individuals without IBS considering the DHEA or DHEA-S level, the present study did not show a lower DHEA-S response to prolonged stress by individuals with IBS, unlike the response in acute stress; therefore, DHEA-S level in IBS could be affected by the type of stress.

There are two distinct strengths in this study. First, this is one of a limited number of studies on both DHEA and DHEA-S focused on individuals with IBS. Although the DHEA-S level did not show a significant effect of IBS or any interactions in the present study, assessment of not only cortisol but also DHEA and the calculation of the cortisol/DHEA ratio could help clarify the underlying cause of aggravation of IBS symptoms, considering the psychological influence and the effect of DHEA on immunological function in a manner opposite to that of cortisol. By determining DHEA or DHEA-S, an effect of cortisol under psychological stress in individuals with IBS was clearly revealed, both under prolonged stress and acute stress [15], while the previous study results showed that DHEA-S secretion differed in individuals with IBS between prolonged-stress and acute-stress situations. Second, this study clarified the physiological characteristics of individuals with IBS under prolonged stress. Although the relationship between the HPA axis, immune system, and abdominal symptoms has been reported previously, the roles of adrenal hormones in individuals with IBS under prolonged stress have not yet been clarified. The present study could offer new insights into future IBS-related research.

On the other hand, there are several limitations to the present study. First, although we found a high cortisol/DHEA ratio 30 min after awakening in individuals with IBS, we did not determine how these ratios changed during the period of time between awakening and bedtime. Second, all participants of the present study were young female students. Future study should investigate both female and male participants. It has been reported that the associations of psychosocial stress with endocrine activities differed by gender (e.g., Weekes et al., [44]). Gender differences in the psychological functioning of patients with IBS have also been reported (Tang et al., [45]). Third, the present study had no control groups because it is mandatory for all the students in the college we investigated to participate in the teaching practice. Fourth, the number of participants in the present study was relatively small. Fifth, sleeping hours and awakening time differed across the four time points. In a previous study [46], later awakening time was associated with a lower cortisol awakening response. This should be considered, although the effects of awakening time were statistically adjusted.

## Conclusion

The cortisol/DHEA ratio of the participants of the present study responded to prolonged stress, and individuals with IBS showed a higher cortisol/DHEA ratio at 30 min after awakening than individuals without IBS during the experimental period. Both the results of a previous study [15] and the present study—lower DHEA-S level under acute stress and higher cortisol/DHEA ratio under prolonged stress in individuals with IBS—may indicate cortisol dominance under stressful situations in IBS.

## Abbreviations

IBS: Irritable bowel syndrome
HPA: Hypothalamic–pituitary–adrenal
CRH: Corticotropin-releasing hormone
ACTH: Adrenocorticotrophic hormone
DHEA: Dehydroepiandrosterone
DHEA-S: Dehydroepiandrosterone sulfate
RMQ: Rome II modular questionnaire
PSS: Perceived stress scale
